# State of the Science: Biologically Based Modeling in Risk Assessment

**DOI:** 10.1155/2012/758182

**Published:** 2012-11-28

**Authors:** Jane C. Caldwell, Kannan Krishnan, Marina V. Evans

**Affiliations:** ^1^National Center for Environmental Assessment, Office of Research and Development, US Environmental Protection Agency, Washington, DC 20460, USA; ^2^Department of Occupational and Environmental Health, Faculty of Medicine, Université de Montréal, Montreal, QC, Canada H3T 1A8; ^3^National Health and Environmental Effects Research Laboratory, Office of Research and Development, US Environmental Protection Agency, Research Triangle Park, NC 27711, USA

The health risk assessment from exposure to a particular agent is preferred when the assessment is based on a relevant measure of internal dose (e.g., maximal concentration of an active metabolite in target tissue) rather than simply the administered dose or exposure concentration. To obtain such measurements, the relevant biology, physicochemical properties, and biochemical mechanisms of a specific agent are used to construct biologically based models that can be used to predict its uptake, disposition, target tissue dose, and ensuing tissue responses in test animals and humans. The focus of this special issue is the state of the science underlying the development and application of a specific type of biologically based model (i.e., physiologically based pharmacokinetic or (PBPK) models) in risk assessment. The fourteen papers presented herein address critical issues and advances relating to their use in current risk assessment approaches with a focus on their use in emerging toxicology paradigms as well. 

The first paper in this issue (J. C. Caldwell et al.) presents an overview that (1) briefly introduces the papers contained in this special issue; (2) provides context for how they inform best modeling practices and state-of-the-art risk assessment applications of PBPK models; (3) discusses limitations and bridges of modeling approaches for future applications and how papers within the issue fit into that emerging science. Specifically, novel approaches for estimation/characterization of metabolic parameters for PBPK models are examined in two articles (i.e., W. S. Cuello et al. and T. Peyret and K. Krishnan) with application of PBPK models for determination of exposure and interpretation of biomarker data examined in three articles (i.e., R. A. Becker et al., C. Lu and Andres, and K. McNally et al.). The special issue contains several case studies that illustrate the development and/or use of quantitative models to address specific needs of risk assessment of selected chemicals (i.e., H. Mielke and U. Gundert-Remy, M. D. Taylor et al., D. C. Dorman et al., and C. Huynh-Delerme et al.) and chemical mixtures (N. C. Y. Wang et al. and A. F. Sasso et al.). The impact of human population subgroup variability on the magnitude of uncertainty factor developed as part of risk assessment of volatile organic chemicals is also described (i.e., M. Valcke et al.). A broader perspective on the use of PBPK models for addressing contemporary issues in risk assessment is presented along with a progress report on the development of a PBPK “tool-box” for more general applications (i.e., M. Mumtaz et al.). 

In summary, the collection of papers by leading experts in this field that comprise this special issue provides insight and tools for a wide spectrum of risk assessment applications. These papers can be used for (1) facilitation of the screening and prioritization of chemicals, (2) linkage of exposure to internal dose, (3) analysis of mechanistic information and prediction of risk to chemical mixtures, and (4) provision of computational techniques and tools to address uncertainty and variability questions related to identification and prediction of responses and pharmacokinetics in potentially sensitive subpopulations. We hope that this “snapshot” of the current state of the science, best practices, and challenges for application of these tools in emerging risk assessment approaches and data will be of tremendous interest to the regulatory and scientific communities internationally. This information can build a foundation for use of these predictive models for a variety of applications and for future reduction of uncertainty in those predictions. 

